# Fundus Autofluorescence in Posterior and Panuveitis—An Under-Estimated Imaging Technique: A Review and Case Series

**DOI:** 10.3390/biom14050515

**Published:** 2024-04-25

**Authors:** Matthias M. Mauschitz, Markus Zeller, Pradeep Sagar, Suchitra Biswal, Gabriela Guzman, Jan H. Terheyden, Carsten H. Meyer, Frank G. Holz, Carsten Heinz, Uwe Pleyer, Robert P. Finger, Maximilian W. M. Wintergerst

**Affiliations:** 1 Department of Ophthalmology, University Hospital Bonn, University of Bonn, 53127 Bonn, Germany; 2 Sankara Academy of Vision, Sankara Eye Hospital Shimoga, Shimoga 577202, India; pradeepsagarbk@gmail.com (P.S.);; 3 Augenzentrum Grischun, 7000 Chur, Switzerland; 4 Department of Ophthalmology, Philipps University, 35037 Marburg, Germany; 5 Department of Ophthalmology, St. Franziskus-Hospital Muenster, 48145 Muenster, Germany; carsten.heinz@augen-franziskus.de; 6 Department of Ophthalmology, University Duisburg-Essen, 45122 Essen, Germany; 7 Charité-Universitätsmedizin Berlin, Corporate Member of Freie Universität Berlin and Humboldt-Universität zu Berlin, 13353 Berlin, Germany; uwe.pleyer@charite.de; 8 Department of Ophthalmology, Berlin and Berlin Institute of Health, 13353 Berlin, Germany; 9 Department of Ophthalmology, University Medical Center Mannheim, Heidelberg University, 68167 Mannheim, Germany

**Keywords:** biomarker, FAF, fundus autofluorescence, inflammation, uveitis

## Abstract

Fundus autofluorescence (FAF) is a prompt and non-invasive imaging modality helpful in detecting pathological abnormalities within the retina and the choroid. This narrative review and case series provides an overview on the current application of FAF in posterior and panuveitis. The literature was reviewed for articles on lesion characteristics on FAF of specific posterior and panuveitis entities as well as benefits and limitations of FAF for diagnosing and monitoring disease. FAF characteristics are described for non-infectious and infectious uveitis forms as well as masquerade syndromes. Dependent on the uveitis entity, FAF is of diagnostic value in detecting disease and following the clinical course. Currently available FAF modalities which differ in excitation wavelengths can provide different pathological insights depending on disease entity and activity. Further studies on the comparison of FAF modalities and their individual value for uveitis diagnosis and monitoring are warranted.

## 1. Introduction

Uveitis is an inflammatory disease spectrum of the uvea, which is the vascularized pigmented layer between the inner retina and the sclera. Depending on the inflamed anatomical structure, this condition can be stratified into the subtypes anterior, intermediate, posterior, and panuveitis. Various posterior and panuveitis entities can be visualized using fundus autofluorescence (FAF).

FAF is an imaging modality that allows a topographic visualization of the distribution of not only lipofuscin (LP) in the retinal pigment epithelium (RPE) cell monolayer but also of other intrinsic fluorophores that may be a result of pathologic processes affecting the outer retina and/or the subneurosensory space [1,2]. It is a diagnostic tool to identify and characterize various retinal diseases using the autofluorescent properties of LP when excited with short-to-medium wavelengths of light. Limitations of FAF are poor visualization in case of media opacity (cataract, vitreous opacities), an often-uncomfortable image acquisition for the patient (due to glare), and very limited options for quantification of the image signal. Depending on the excitation wavelength, FAF modalities can in general be classified as blue-light (BAF), which can be subdivided in short- (swBAF) and long-wavelength blue-light autofluorescence (lwBAF), green-light (GAF), or infrared fundus autofluorescence (IRAF). Blue- and green-light FAF signals primarily originate from LF, while IRAF signal is thought to primarily originate from melanin. In healthy eyes, the LF distribution is higher in the parafoveal area and decreases towards the periphery [2]. For melanin, which is present both in the RPE as well as the choroid, the concentration in the RPE decreases from the periphery to the posterior pole (with an increase again in the macular area) [3]. Choroidal melanin increases from the periphery to the posterior pole [3]. Blue and green light FAF modalities provide information about the metabolic state of the photoreceptor (PR)/RPE complex. An accumulation or reduction in LF quantity can be—among other findings—an early indicator of (degenerative) retinal diseases. Further, it results in an increased FAF signal, whereas a reduction in LF quantity decreases the FAF signal. Distribution patterns of melanin in the RPE and the choroid can change in retinal and choroidal disease; however, underlying pathophysiology remains to be determined. Depending on the excitation wavelength, lesions in different depths and, thus, uveitis entities can be imaged due to altered autofluorescent properties of LF in case of PR/RPE damage. Additionally, minor fluorophores including advanced glycation end products (AGE), flavin adenine dinucleotide, and collagen/elastin might contribute to the altered FAF signal in posterior and panuveitis [4,5].

For this review, we divided these disorders into posterior and panuveitis subclassified by etiology as non-infectious or infectious and masquerade syndromes (Table 1).

Non-infectious uveitis entities that can be imaged using FAF are mainly so-called white dot syndromes and include acute posterior multifocal placoid pigment epitheliopathy (APMPPE), multiple evanescent white dot syndrome (MEWDS), multifocal choroiditis and panuveitis (MCP), punctate inner choroidopathy (PIC), serpiginous choroiditis, and acute zonal occult outer retinopathy (AZOOR). Additionally, FAF can be helpful in patients with birdshot chorioretinopathy (BSCR), Vogt–Koyanagi–Harada (VKH) disease, and Behçet Uveitis. Infectious uveitis entities that can be imaged using FAF include cytomegalovirus (CMV) retinitis, syphilis, tuberculosis, acute retinal necrosis (ARN), and progressive outer retinal necrosis (PORN). Additionally, the masquerade syndromes such as intraocular lymphoma and choroidal melanoma can be imaged using FAF.

This review gives an in-depth overview on the current application of FAF in aiding diagnosing and monitoring different uveitis entities. In previous uveitis imaging reviews, FAF findings are often only briefly mentioned, as FAF may be considered only an additional imaging modality rather than a reliable, quick, and non-invasive imaging technology itself which can aid uveitis diagnosis and monitoring.

## 2. Materials and Methods

PubMed was searched in January 2023 for relevant literature regarding FAF in posterior uveitis. The search term was: ((“uveit*” [Title] OR “birdshot*” [Title] OR “choroiditis*” [Title] OR “koyanagi*” [Title] OR “placoid pigment” [Title] OR “APMPPE” [Title] OR “acute retinal necrosis” [Title] OR “progressive outer retinal necrosis” [Title] OR “punctate inner” [Title] OR “pigment epitheliopathy” [Title] OR “pigment epitheliopathies” [Title] OR “white dot” [Title] OR “inflammatory eye disease” [Title] OR “inflammatory eye diseases” [Title] OR “vitritis” [Title] OR “Acute Zonal Occult” [Title] OR “AZOOR” [Title] OR “Melanoma” [Title/Abstract] OR “Lymphoma” [Title/Abstract] OR ((((“retinitis*” [Title] NOT “pigmentosa*” [Title]) NOT “punctata” [Title]) NOT “sclopetaria” [Title]) NOT “solar” [Title]) OR ((“vasculitis*” [Title] OR “sarcoidos*” [Title] OR “behçet*” [Title] OR “behcet*” [Title]) AND (“eye” [Title] OR “ophthalm*” [Title] OR “retina*” [Title] OR “retinop*” [Title] OR “choroid*” [Title] OR “choriocap*” [Title] OR “optic nerve” [Title] OR “ophthalmi*” [Title] OR “ocular*” [Title]))) AND “fundus*” [Title] AND “autofluorescen*” [Title]) OR (“behcet” [Title] AND “fundus*” [Title/Abstract] AND “autofluorescen*” [Title/Abstract]).

We found 75 potentially relevant publications. The abstracts of all obtained articles were screened for eligibility. Additionally references of key articles were also included. Exclusion criteria were comments and letters to the editor, articles with non-English abstracts as well as articles without relevance for this review. We selected the below-mentioned specific forms of uveitis based on the available literature, which is sparse or even non-existing for various very rare uveitis conditions. Retinal images of the figures included in this review include color fundus photography (CFP, Eidon, CenterVue, Padua, Italy), swBAF (Spectralis, Heidelberg Engineering, Heidelberg, Germany, excitation wavelength 450 nm), lwBAF (Spectralis, Heidelberg Engineering, Heidelberg, Germany, excitation wavelength 488 nm), GAF (Spectralis, Heidelberg Engineering, Heidelberg, Germany, excitation wavelength 518 nm), and IRAF (Spectralis, Heidelberg Engineering, Heidelberg, Germany, excitation wavelength 787 nm), and are from patients recruited from uveitis and other outpatient clinics at the department of ophthalmology at the University of Bonn, Germany. All subjects gave their informed consent after explanation of the nature and possible consequences of the study prior to being included. Ethics approval was obtained by the ethics committee at the University of Bonn (ethics approval ID 011/18), and the study was conducted in adherence to the Declaration of Helsinki.

## 3. Results

### 3.1. Non-Infectious Uveitis

#### 3.1.1. Acute Posterior Multifocal Placoid Pigment Epitheliopathy (APMPPE)

An active uveitis is often associated with hyperautofluorescence in FAF; however, APMPPE is an exception, in which hypoautofluorescence represents the onset phase of the disease [6]. The hypoautofluorescence in active APMPPE is considered a result of signal blockage due to RPE cell edema. However, an inactive disease and chorioretinal atrophy in APMPPE also present with hypoautofluorescence. These findings were also seen using GAF (585 nm excitation wavelength) imaging [7].

**Figure 1:**
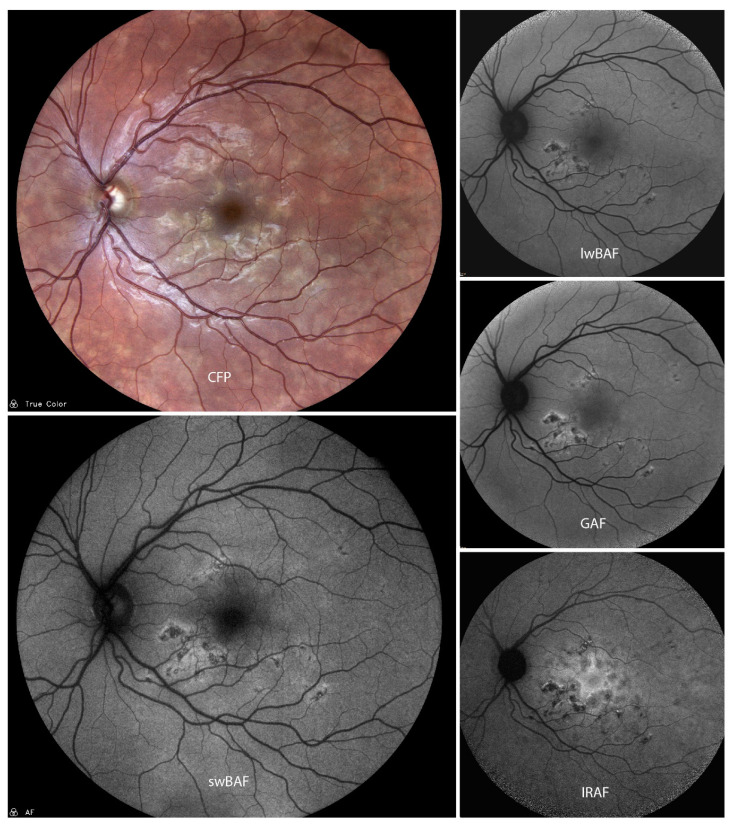
A case of acute posterior multifocal placoid pigment epitheliopathy (APMPPE) on color fundus photography (CFP), short-wavelength blue-light autofluorescence (swBAF, 450 nm), long-wavelength blue-light autofluorescence (lwBAF, 488 nm), green-light autofluorescence (GAF, 518 nm) and infrared-light autofluorescence (IRAF, 787 nm). The typical FAF pattern with hypoautofluorescent lesions with hyperautofluorescent borders is illustrated on different FAF modalities. Source: [13].

Characteristic features of APMPPE lesions on FAF (Figure 1) include well-demarcated hypoautofluorescent lesions with edges that are hyperautofluorescent [8,9]. FAF also shows acute inflammation of the RPE, which can lead to long-term damages such as RPE atrophy. The extension and localization of these hypoautofluorescent spots can be clearly defined by autofluorescence measures and may be important for prognosis of visual acuity [8,9].

In mild APMPPE cases, FAF may only show hyper- and no hypoautofluorescent lesions during the acute phase. The hyperautofluorescent lesions are hypothesized to indicate swelling of RPE cells [10]. During the recovery phase, a disappearance of hyperautofluorescent lesions can indicate damage to the RPE cells [10]. Therefore, FAF can aid in evaluating APMPPE disease stage and severity. During the course of disease, lesions showing hyperautofluorescence may change into hypoautofluorescence indicating scar formation [11].

The combination of FAF with funduscopy or another non-invasive imaging method such as optical coherence tomography (OCT) or OCT angiography can be helpful for the diagnosis of APMPPE without using invasive methods, i.e., fluorescein or indocyanine green angiography [6,12].

#### 3.1.2. Multiple Evanescent White Dots Syndrome (MEWDS)

In MEWDS, FAF can demonstrate early and late hyperautofluorescence of the white dots [14,15]. The FAF features of MEWDS vary between the different wavelengths. Using a long-wavelength blue-light autofluorescence with 488 nm excitation wavelength, the site of white dots present hyperautofluorescence [16]. Blue-light FAF provides irregular speckled patterns without a precise identification of the single inflammatory lesions [17,18]. NIR-FAF (787 nm excitation wavelength) is characterized by hypoautofluorescent spots corresponding to the inflammatory lesions [17,18]. NIR-FAF and retro mode imaging cannot be affected by blood flow variation, and therefore offer a good-quality visualization of lesions typical in MEWDS [17]. Retro mode imaging allows a pseudo-3D reconstruction of the retina and choroid via scanning laser ophthalmoscopy using an infrared laser and a modified central stop. In the sparse existing literature on further modalities with excitation wavelengths of 532 nm and 633 nm, authors report diffuse hyperautofluorescent appearing lesions in the posterior pole with surrounding hyperautofluorescent spots outside the vascular arcades [19].

Ultra-wide-field FAF imaging also enables an improved visualization of the affected lesions with even a greater sensitivity than conventional color fundus photography [19]. An ultra-wide-field FAF of 200 degrees can offer an advantage when evaluating pan-fundus lesions [19].

In comparison to fluorescein angiography (FA), hyperautofluorescent spots on FAF are brighter and show a greater contrast [20]. FAF can represent a useful non-invasive method for the diagnosis, tracking, and control of MEWDS, in addition to invasive methods such as FA and indocyanine green-angiography (ICG-A) [20].

Moreover, FAF also suggests the inflammatory nature of the disease due to the perturbation of the photoreceptor–RPE complex, which affects RPE health and integrity [14].

#### 3.1.3. Multifocal Choroiditis and Panuveitis (MCP)

**Figure 2:**
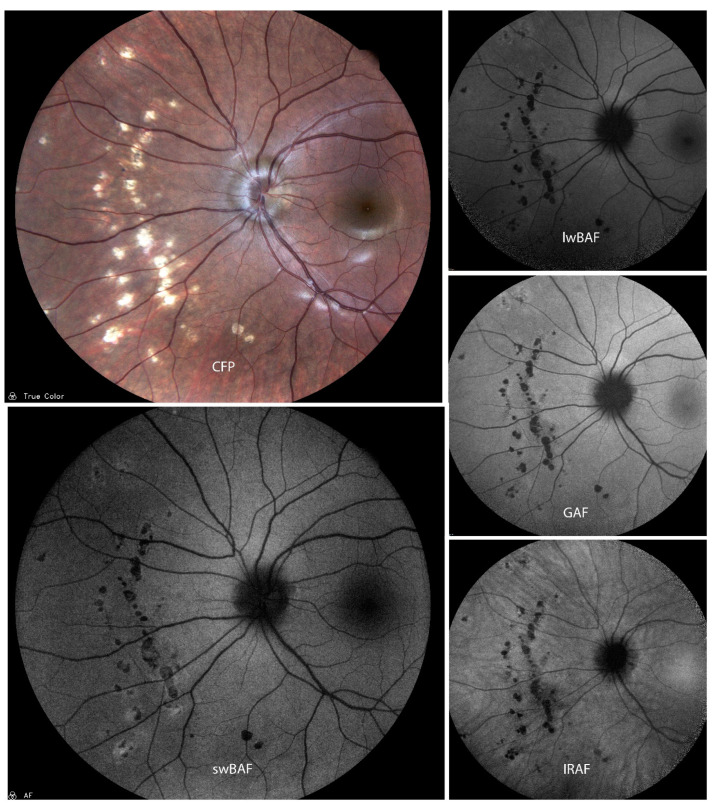
A case of inactive presumed multifocal choroiditis and panuveitis (MCP) on color fundus photography (CFP), short-wavelength blue-light autofluorescence (swBAF, 450 nm), long-wavelength blue-light autofluorescence (lwBAF, 488 nm), green-light autofluorescence (GAF, 518 nm), and infrared-light autofluorescence (IRAF, 787 nm). Bright atrophic MCP lesions are mainly hypoautofluorescent on different FAF modalities. Source: [13].

GAF (535–585 nm excitation wavelength) can provide information about inflammatory damage and secondary macular neovascularizations (MNVs) in MCP (Figure 2). Two main types of hypoautofluorescence can be distinguished [21]: larger hypoautofluorescent spots, greater than 125 microns, which are also visible on CFP, and smaller hypoautofluorescent spots measuring less than 125 microns, which are rarely visible on CFP.

These smaller spots are commonly found in the peripapillary and macular regions and not all of them are visible on FA [21]. Moreover, FAF can aid in imaging new or enlarging spots during clinical course and also help establish a diagnosis of MCP in doubtful cases [21]. The extent of MNVs can also be imaged by FAF and a combination with other non-invasive methods such as optical coherence tomography (OCT) can reduce the need for using invasive imaging methods such as FA and ICG-A [21].

However, FAF should not be considered a substitute of ICG-A, but rather as a non-invasive complementary diagnostic and prognostic tool to achieve a more detailed disease characterization. Once diagnosis has been made, FAF imaging can be used as a non-invasive follow-up tool in addition to other modalities such as OCT [22].

#### 3.1.4. Punctate Inner Choroidopathy (PIC)

**Figure 3:**
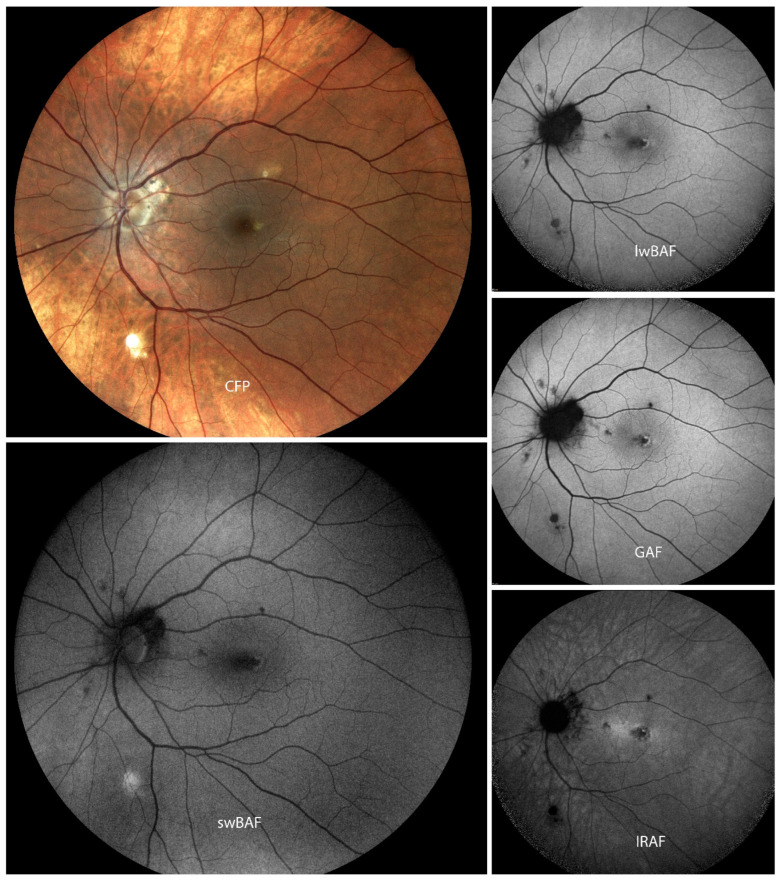
A case of punctate inner choroidopathy (PIC) with parafoveal macular neovascularization on color fundus photography (CFP), short-wavelength blue-light autofluorescence (swBAF, 450 nm), long-wavelength blue-light autofluorescence (lwBAF, 488 nm), green-light autofluorescence (GAF, 518 nm), and infrared-light autofluorescence (IRAF, 787 nm). While the CFP shows some of the lesions in PIC, different FAF modalities can aid in detection of more lesions. Source: [13].

FAF is a non-invasive technique that can aid diagnosis, management, and monitoring of PIC (Figure 3). lwBAF (488 nm excitation wavelength) and GAF (580 nm excitation wavelength) help to visualize and delineate damaged RPE [23] and anatomic disruption of the photoreceptor–RPE complex [24]. Substantial differences between lwBAF (488 nm) and IRAF (787 nm) were not seen; however, subclinical lesions were more distinctive on IRAF [25]. GAF (585 nm excitation wavelength) may also be used to predict the response and/or future resistivity to treatments and can be used alongside FA or OCT to monitor the activation and stabilization of PIC [26]. FAF aids in imaging symptomatic acute PIC, which is manifested as hyperautofluorescence and ellipsoid zone (EZ) loss [27] on OCT, and can also be a helpful imaging technique to visualize long-term sequelae of previously active lesions, even after years of inactivity [23]. In addition, FAF may be used in imaging the resolution of many peripapillary lesions that correspond to the normalization of the visual field defect on subsequent visual field testing and blind spot enlargement [23]. It has been shown that FAF yields information that could not be imaged with other techniques [23,28]. FAF imaging of patients with PIC has revealed extensive pathology beyond areas that appeared clinically unaffected [24]. Moreover, FAF imaging can also help tracking the evolution of PIC lesions and identify combined MEWDS or AZOOR lesions [25]. FAF can be used as a complementary imaging technique to (SD-)OCT and fluorescein angiography for the monitoring of PIC [25]. Peripheral FAF abnormalities were detected despite the notable absence of these lesions with clinical examination and color fundus photography. These FAF findings were important in guiding clinical follow-up and response to therapy. The FAF lesions resolved with treatment [29].

#### 3.1.5. Serpiginous Choroiditis (SC)

**Figure 4:**
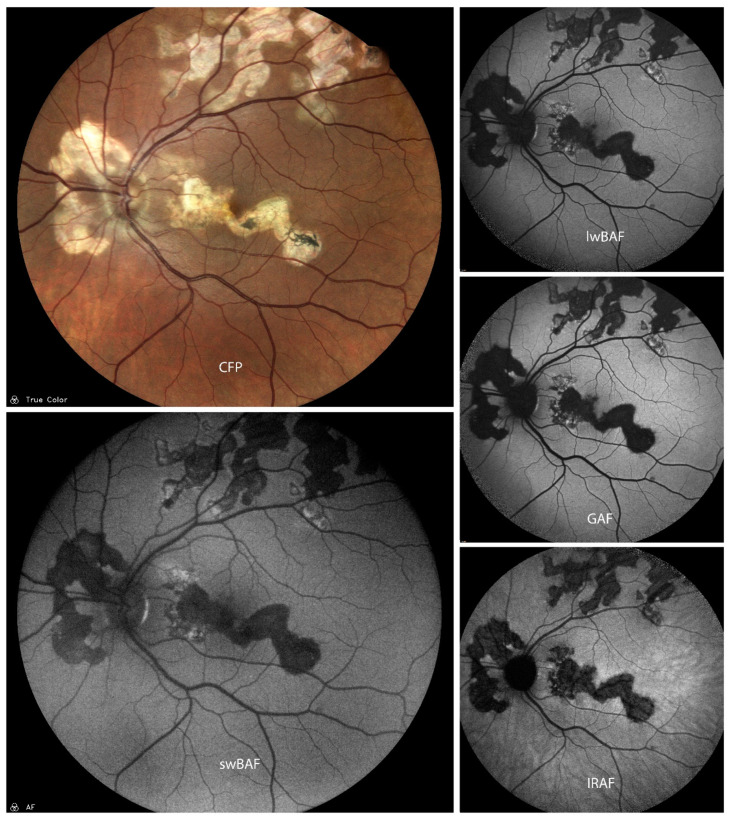
A case of active serpiginous choroiditis on color fundus photography (CFP), short-wavelength blue-light autofluorescence (swBAF, 450 nm), long-wavelength blue-light autofluorescence (lwBAF, 488 nm), green-light autofluorescence (GAF, 518 nm), and infrared-light autofluorescence (IRAF, 787 nm). Inactive lesions are completely hypoautofluorescent on FAF modalities, while active lesion borders depict a speckled pattern of hyper- and hypoautofluorescence. Source: [13].

FAF is a sensitive imaging technique for detecting RPE changes such as activity and recovery in acute episodes of SC (Figure 4) [30]. It can also be helpful for the evaluation of activity of SC as FAF shows hyperautofluorescence in the active stages of the disease and a progressive decrease in autofluorescence during the scarring phase [31]. There is a pattern of FAF during the entire course of SC lesions, i.e., during evolution, progression, and healing of SC. During the initial stage of evolution, the lesion is hyperautofluorescent. Faint hyperautofluorescence extending over a large area can predict the future extent of the lesion and might be representative of the actual extent of RPE involvement [32]. FAF may even predict future evolution of lesions in the initial stage [32]. If lesion borders are mainly hyperautofluorescent, it indicates an advancing lesion, which can be clinically essential to predict progression [32]. A lesion becoming inactive shows a sharpening of hyperautofluorescent borders followed by decreased hyperautofluorescence on FAF. These are early signs before the healing border becomes hypoautofluorescent [32]. These findings were similar to the findings in GAF (585 nm) [33].

A combination of FAF and OCT can provide information regarding the extent of RPE and photoreceptor involvement in new lesions [30] and help in monitoring their change during the course of disease [31]. FAF allows an easy identification of recurrences and (SD-)OCT provides information regarding the extent of choroid, RPE, and outer retinal involvement [31]. This combination could be a more sensitive non-invasive imaging technique alternative to angiography [30].

#### 3.1.6. Acute Zonal Occult Outer Retinopathy (AZOOR)

**Figure 5:**
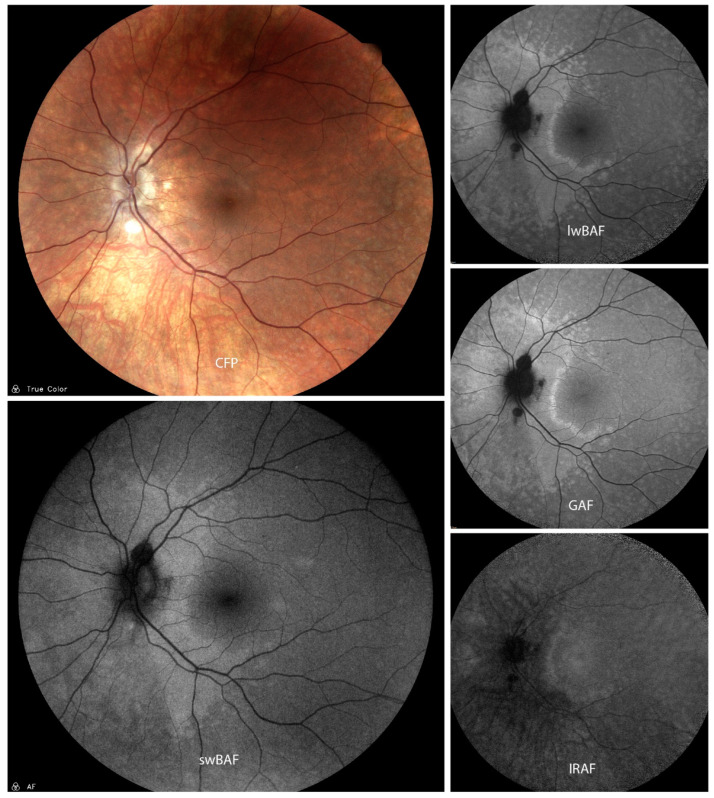
A case of presumed active atypical acute zonal occult outer retinopathy (AZOOR) on color fundus photography (CFP), short-wavelength blue-light autofluorescence (swBAF, 450 nm), long-wavelength blue-light autofluorescence (lwBAF, 488 nm), green-light autofluorescence (GAF, 518 nm), and infrared-light autofluorescence (IRAF, 787 nm). While it is difficult to detect lesions on CFP, they can easily be visualized on different FAF modalities as a hyperautofluorescent pattern expanding from the papilla on swBAF, lwBAF, and GAF, and as a hypoautofluorescent area on IRAF. Source: [13].

lwBAF (488 nm excitation wavelength) and GAF (550 nm excitation wavelength) abnormalities can be detected in in most eyes with AZOOR (Figure 5) [34,35,36]. However, not all eyes diagnosed with AZOOR showed abnormal FAF patterns. The presence of normal FAF imaging cannot exclude a diagnosis of AZOOR completely and additional investigations including mfERG and visual fields may be needed [34].

OCT findings in AZOOR include thinning of the photoreceptor cell layer with loss of the outer segments and abnormal inner retinal lamination [36]. Eyes with AZOOR can show on FAF a progression of hypoautofluorescence area during their follow-up, which can be used for disease monitoring [35,36]. There is a strong correlation between lesions seen on UWFFAF, perimetric, OCT, and ERG. There is a high degree of correlation between (UWF-)FAF findings and perimetry and either full-field-ERG or multi-focal-ERG, depending on the involved retinal zone [37]. There is also a good correlation between (UWF-)FAF and (SD-)OCT macular abnormalities [37]. Additionally, UWFFAF appears to be useful in imaging even more extensive involvement than standard or regular WF-FAF [37]. UWFFAF in combination with SD-OCT can be useful tools in the initial evaluation, detection of the full extent of lesions, and monitoring of progression of AZOOR [37].

#### 3.1.7. Birdshot Chorioretinopathy (BSCR)

GAF (560 nm excitation wavelength) can be important for evaluation and management of BSCR, particularly for monitoring of RPE atrophy. GAF (560 nm excitation wavelength) can reveal RPE atrophy, which may not be visible in other imaging modalities or CFP [38], not only in areas of hypopigmented lesions [39]. Especially in eyes with light fundus pigmentation, lesions can be identified more precisely [38] and more diffuse areas of disease involvement may be detected than clinically [6]. The hypoautofluorescence on GAF (532 nm excitation wavelength) correlates with the duration of the disease and the degree of inflammation in the affected eye [40]. There has been evidence that lwBAF (488 nm excitation wavelength) abnormalities progress from granular to confluent hypoautofluorescence and also that BSCR progresses centripetally towards the fovea [41]. lwBAF (488 nm excitation wavelength) can be used as an imaging tool to monitor patients in terms of disease progression during treatment [41]. Some data suggest that a larger number of choroidal lesions can be identified using a laser with 635 nm excitation wavelength compared to 532 nm [42]. Interestingly, macular hyperfluorescent lesions are not associated with worse LogMAR visual acuity [43]. Also, spectrally resolved autofluorescence imaging can aid in diagnosing BSCR as distinct from similar posterior uveitis entities utilizing the ratio between green and red emission fluorescent components [44].

#### 3.1.8. Vogt–Koyanagi–Harada (VKH) Disease

Peripapillary atrophy, atrophic, and pigmented scars on CFP all show a decreased autofluorescence signal on FAF corresponding with atrophy of the RPE and the outer Retina in eyes with VKH disease [45]. Patches, strands, or irregular pigmentation as well as cystoid macular edema on CFP were associated with increased autofluorescence signal [45].

lwBAF (488 nm excitation wavelength) and NIR-FAF (789 nm excitation wavelength) demonstrate similar patterns when imaging eyes with acute VKH disease, but the patterns might be more evident in NIR-FAF, which originates from the RPE and is not blocked by the macular pigment [46]. Therefore, NIR-FAF could represent an alternative imaging method for an early detection of RPE abnormality, not only in VKH disease but also other posterior diseases [46].

**Figure 6:**
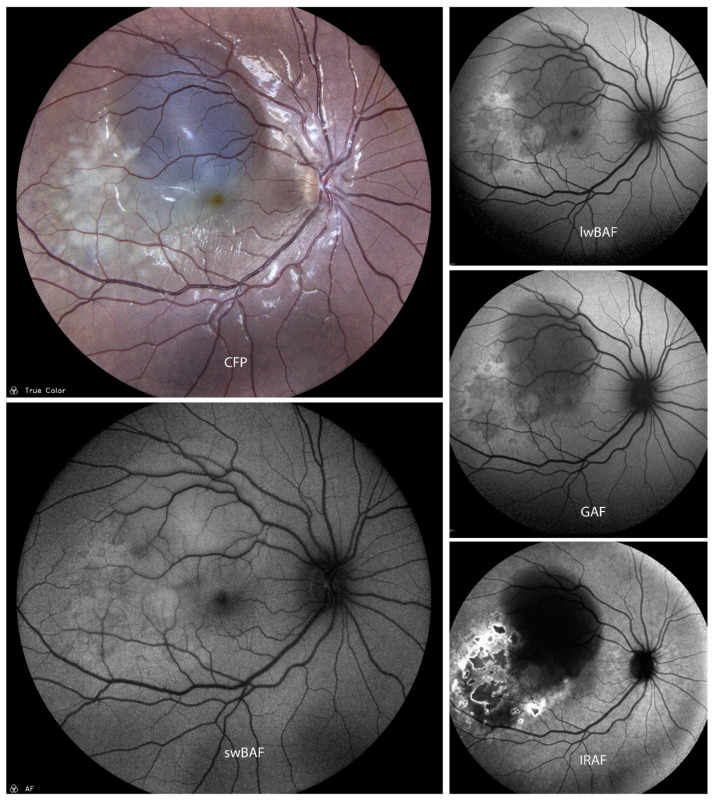
A case of active Vogt–Koyanagi–Harada (VKH) disease on color fundus photography (CFP), short-wavelength blue-light autofluorescence (swBAF, 450 nm), long-wavelength blue-light autofluorescence (lwBAF, 488 nm), green-light autofluorescence (GAF, 518 nm), and infrared-light autofluorescence (IRAF, 787 nm). Lesions are more distinct on IRAF as compared to the other imaging modalities. Source: [13].

In comparison to FA and ICG-A, FAF could even be a superior indicator of the functional status of the RPE in VKH disease (Figure 6), because FA and ICG-A only represent indirect consequences of RPE damage on the kinetics of the dye [45]. A combined use of FAF and SD-OCT could offer an advantage over FA and ICG-A, providing information about the status of the RPE and outer retina in patients with chronic VKH disease. This combined imaging method allows an accurate observation of the extent and severity of RPE and/or outer retinal changes [45].

#### 3.1.9. Behçet Uveitis

Very limited literature is available on FAF in Behçet uveitis. The degree of alteration of RPE can be detected on FAF with consecutive change in lipofuscin distribution, rather than on CFP. Mesquida et al. evaluated 34 eyes of 18 patients with Behçet uveitis and reported multifocal hypoautofluorescent and hyperautofluorescent spots as well as hypofluorescent lesions along the retinal vessels seen on GAF (532 nm excitation wavelength) imaging [47]. The authors suggested that the active retinal vasculitis results in alteration of RPE, leading to FAF abnormalities which are better visible on wide field FAF imaging. FAF provides more specific information regarding the number of lesions and extent of retinal involvement compared to CFP. Mesquida et al. concluded that FAF adds to color fundus imaging in evaluating and monitoring the disease [47].

#### 3.1.10. Ocular Sarcoidosis

Ocular involvement is observed in about 30–50% of patients with sarcoidosis [7]. Inflammatory lesions involving RPE can present as hyperautofluorescent, while healed lesions can present as hypoautofluorescence due to atrophy of the RPE and the overlying photoreceptors [7,44,48]. These findings, observed on swBAF (450 nm excitation wavelength), corresponded to choroidal thinning on OCT and white dots in CFP [44]. Lesions showed a predominant green emission wavelength when compared to red emission wavelength of low visibility on intensity-normalized Color-FAF [44].

Moreover, wide-field imaging techniques have been suggested to help study the full extent of ocular sarcoidosis lesions on FAF [7]. In a case study, de Saint Sauveur et al. showed that the pre-retinal nodules or sarcoid lesions also appeared as multiple hypoautofluorescent spots on infrared autofluorescence (IRAF) [49]. There are, however, no specific characteristic changes on IRAF images in chronic sarcoidosis [7].

### 3.2. Infectious Uveitis

#### 3.2.1. Cytomegalovirus (CMV) Retinitis

**Figure 7:**
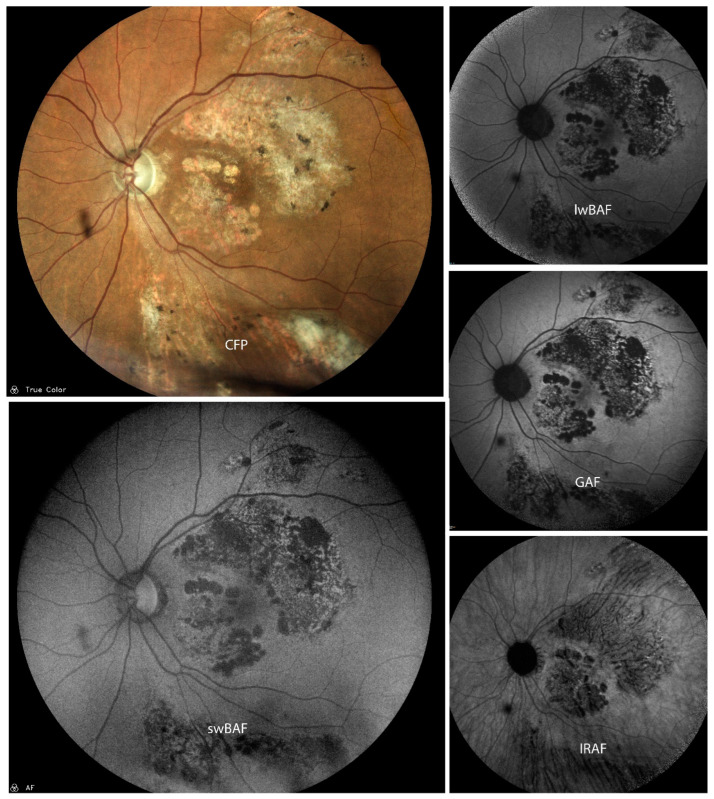
A case of inactive cytomegalovirus (CMV) retinitis on color fundus photography (CFP), short-wavelength blue-light autofluorescence (swBAF, 450 nm), long-wavelength blue-light autofluorescence (lwBAF, 488 nm), green-light autofluorescence (GAF, 518 nm), and infrared-light autofluorescence (IRAF, 787 nm). A mixed pattern of hypo- and hyperautofluorescence is evident on the different FAF modalities. Source: [13].

Few studies have evaluated the role of FAF in CMV retinitis. FAF shows hyperautofluorescence in the active phase of disease which corresponds to the granular lesions (Figure 7). Mixed hyper- and hypoautofluorescence pattern can be seen in the consecutive inactivation phase [50].

Tadepalli et al. reported few advantages of UWF-FAF over UWF fundus photograph including the ability to detect more extensive area of involvement, to delineate the lesions more accurately, to differentiate the disease from other types of retinal lesions, and its ability to detect recurrence during treatment. In this series, nine eyes had recurrence and FAF picked up the recurrence in seven eyes. Recurrence was noted in six eyes at the hyper autofluorescent edge of pre-existing lesions, and in one eye from other location. UWF- FAF had more advantages in more central lesions within vortex veins granular lesions [50].

The disadvantages of UWF-FAF include its inability to detect lesions extending outside of the vortex veins and hemorrhagic lesions are seen as areas of blocked autofluorescence [50].

Yashiro et al. evaluated four eyes with short-wavelength autofluorescence (SW-AF) and near-infrared autofluorescence (IR-AF). In the initial stages of the disease, SW-AF showed hyperautofluorescence and IR-AF showed hypoautofluorescence. In the remission stage, SW-AF showed hypoautofluorescence and IR-AF showed hyperautofluorescence [51]. Yeh et al. evaluated nine eyes with CMV retinitis and reported that FAF shows a hyperautofluorescence signal corresponding to the advancing edge of retinitis. They also noted that the areas of RPE atrophy corresponding to prior retinitis showed stippled areas of hypoautofluorescence and hyperautofluorescence. In three eyes with subtle reactivation of the disease, FAF showed hyperfluorescent borders corresponding to the active edge and was helpful in detection of reactivation. The authors noted diffuse punctate hyperautofluorescence following intravitreal ganciclovir and foscarnet in one case and hypothesized that it may be suggestive of drug toxicity [33].

#### 3.2.2. Syphilis

**Figure 8:**
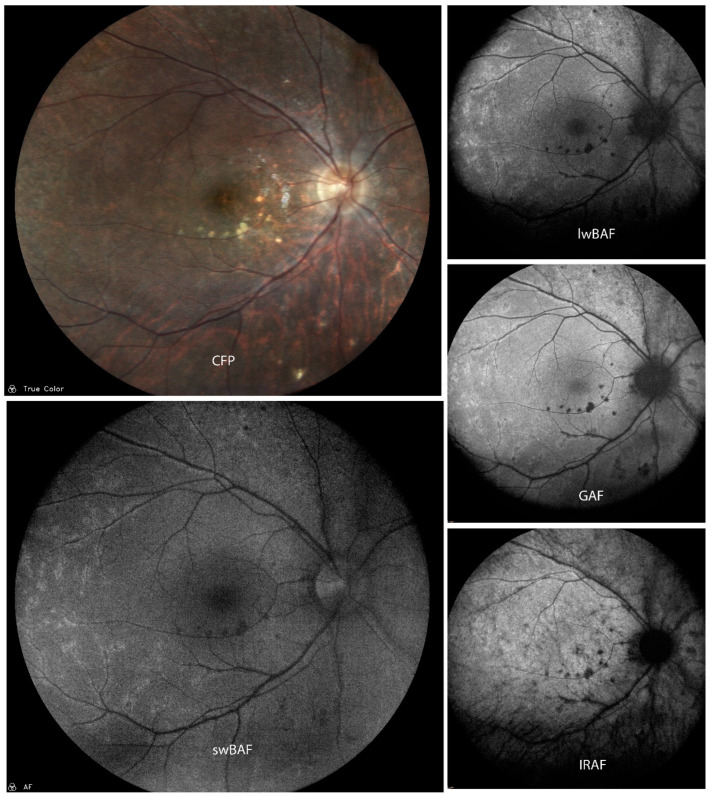
A case of mildly active syphilis on color fundus photography (CFP), short-wavelength blue-light autofluorescence (swBAF, 450 nm), long-wavelength blue-light autofluorescence (lwBAF, 488 nm), green-light autofluorescence (GAF, 518 nm), and infrared-light autofluorescence (IRAF, 787 nm). Multiple hypoautofluorescent lesions on FAF corresponding to white spots on CFP and perivascular hyperautofluorescence are present. Source: [13].

In cases of chronic syphilitic outer retinitis (SOR), (Ultra-Wide-Field)-GAF (532 nm excitation wavelength) shows a similar pattern to the trizonal pattern of AZOOR (Figure 8) [52]. Hence, in cases presenting like AZOOR, SOR should be excluded using laboratory screening. Multimodal imaging using UWF-FAF and SD-OCT can show pathological changes at the level of the outer retina and RPE in SOR. These imaging modalities can be useful for monitoring a response to the treatment of SOR [52].

#### 3.2.3. Acute Retinal Necrosis (ARN)

lwBAF (488 nm excitation wavelength) and GAF (585 nm excitation wavelength) both allow a more accurate delineation of lesions in ARN than in color photos, because FAF grants a visualization of higher contrast of the borders of the lesions [53]. The areas of retinal atrophy show a persistent hypoautofluorescence. The borders of disease activity correlate with a high-contrast change in FAF-patterns, which can be used to better monitor disease progression [53].

#### 3.2.4. Progressive Outer Retinal Necrosis (PORN)

The combination of GAF (580 nm excitation wavelength) and OCT is useful for the characterization of RPE and retinal anatomy in eyes with PORN. This multimodal imaging reveals progressive changes indicative of widespread dysfunction in the RPE and outer retina [54]. FAF changes follow the retinal opacification observed at an early course of the disease. Retinal opacification appearing on FAF is correlated with areas of retinal necrosis and/or tissue breakdown on OCT. With a continuing tissue breakdown and necrosis in the outer retina, lipofuscin accumulates in the RPE, causing a patchy stippled FAF pattern [54].

#### 3.2.5. Tuberculosis

Serpiginous-like choroiditis (SLC) and choroidal granuloma are believed to be related to tuberculosis. Gupta et al. evaluated 36 eyes with presumed tubercular SLC and noted that the GAF (510–580 nm excitation wavelength) pattern changes during the course of the disease [55]. In the acute stage (stage 1), lesions showed an amorphous appearance due to an ill-defined halo of hyperautofluorescence. During the subacute healing phase (stage 2), lesions were surrounded by a thin rim of hypoautofluorescence and showed a stippled pattern of hyperautofluorescence. In the nearly resolved stage (stage 3), lesions showed a stippled pattern of hypoautofluorescence. On complete resolution (stage 4), lesions were uniformly hypoautofluorescent. The authors concluded that FAF is a useful imaging tool for monitoring the lesions in SLC.

In a prospective study, Bansal et al. evaluated four eyes with SLC to correlate the findings of OCT and FAF [56]. The hyperautofluorescent areas in the acute stage of disease corresponded to hyperreflective areas on OCT involving RPE, interdigitation zone, EZ, external limiting membrane, and outer nuclear layer. In the inactivation phase, the lesions showed a hypoautofluorescent border with a hyperautofluorescent center and corresponded to knob-like elevations of outer retina. On complete clinical resolution, the hypoautofluorescent areas corresponded to area of loss of RPE, interdigitation zone, EZ, and external limiting membrane [56].

In a case report, Mishra et al. described a unique finding of dual margins of hyperautofluorescence in a case of SLC with paradoxical worsening following anti-tubercular therapy [57]. The authors postulated that the presence of this finding could indicate increased inflammatory activity, which would result in paradoxical worsening.

### 3.3. Masquerade Syndromes

#### 3.3.1. Intraocular Lymphoma

Vitreoretinal lymphoma (VRL) is a subset of central nervous system lymphoma. It masquerades as chronic intermediate uveitis, and it is temporarily responsive to steroids, which makes it difficult to differentiate from uveitis.

A variable pattern of autofluorescence has been reported in VRL. In a retrospective study, Casady et al. reported a granular hyperautofluorescence pattern in active stage consisting of a hypoautofluorescent ring surrounding the hyperautofluorescence spot in VRL using FAF with 580 nm excitation wavelength [58]. These hyperfluorescent spots correlated to the typical hypofluorescent “leopard” spots on fluorescein angiography. In contrast, Egawa et al. reported the granular autofluorescence pattern on 535–580 nm excitation wavelength FAF consisting of a hyperautofluorescent ring surrounding a hyperautofluorescent spot [59]. Egawa et al. hypothesized that the hyperautofluorescence in VRL is due to accumulation of lipofuscin in RPE cells, adjacent to tumor cells [59]. However, Pantanelli et al. identified the intrinsic autofluorescence on 351 nm, 458 nm, and 488 nm excitation wavelength FAF as a property of malignant B-cells in an in vitro study, which could in part explain the abnormal FAF in VRL [60].

#### 3.3.2. Choroidal Melanoma

Choroidal melanoma can masquerade as intraocular inflammation, masking the underlying tumor. We found no studies that compared FAF in cases with uveitis and choroidal melanoma. Few studies evaluated FAF patterns of choroidal melanoma and choroidal nevus, but findings were contradicting. In contrast to choroidal nevi, choroidal melanoma more frequently shows hyperautofluorescence due to orange pigment. Hypoautofluorescence in choroidal melanoma could be due to RPE atrophy overlying the tumor, recent hemorrhage, or subretinal fluid.

Lavinsky et al. reported in 20 patients that most of the nevi do not present a characteristic pattern of lwBAF (488 nm excitation wavelength), whereas melanomas tend to show hyperautofluorescence with confluent plaque-like configuration and concluded that FAF helps in differentiating the pigmented lesions of the choroid [61]. In contrast, Cennamo G et al. reported, in a case series of 100 choroidal nevi and 65 choroidal melanoma, normal pattern of FAF in 40 patients with choroidal nevi, hypoautofluorescence in 60 patients with choroidal nevi, and plaque-like hyperautofluorescence in 29 patients of choroidal melanoma [62].

Bindewald-Wittich et al. evaluated 31 eyes with choroidal melanoma using blue wave autofluorescence at baseline and after Ruthenium-106 brachytherapy [63]. At baseline, the authors noted laminar hypoautofluorescence due to alterations in RPE overlying the tumor and focal hyperautofluorescence due to orange pigment. The presence of SRF can result in additional FAF findings including hypoautofluorescence due to blockage in cases with recent onset SRF, hyperautofluorescence due to accumulation of fluorophores in the SRF during the course, and hypoautofluorescence due to RPE loss due to persistent SRF in the chronic stage. The FAF patterns in the radiation field following brachytherapy included a rim of hyperautofluorescence at the border of irradiation field and FAF mottling characterized by irregular pattern of hyperautofluorescence with speckled areas of hypoautofluorescence [63,64].

## 4. Discussion

As with many rare diseases, there is a lack of sufficient evidence from controlled randomized clinical trials regarding clinical management including diagnostic work-up and therapy of posterior and panuveitis [65,66]. One prerequisite for this are objective and reliable biomarkers and endpoints for routine clinical practice and clinical trials. However, we largely lack such endpoints for uveitis, as inflammation and complications are mostly graded using subjective, relatively unreliable clinical scales. This review demonstrates that FAF may represent an objective method to qualitatively and quantitatively monitor (chorio)retinal lesions in posterior and panuveitis.

FAF can aid in monitoring extend of (chorio)retinal lesions and in differentiation of active and inactive disease depending on autofluorescence modality in a variety of posterior and panuveitis entities [18,22,35,67,68,69,70,71,72]; hyperautofluorescence is often associated with active disease, which is suggested to be associated with an increase in fluorophores like LF within the RPE, e.g., during an acute inflammatory phase. The detailed nature of involved fluorophores remains unclear and more research on this is warranted. In contrast, hypoautofluorescence is often correlated with inactive disease and chorioretinal atrophy. Furthermore, the pattern of FAF changes when lesions evolve from an acute stage to recovery.

Distinction of different posterior and panuveitis entities can be challenging, though imperative to initiate the correct diagnostic and therapeutic decision as well as to determine the appropriate monitoring interval. As outlined by this review, uveitis entities can show a distinctive pattern of autofluorescence in terms of morphology and autofluorescence characteristics (extent and configuration of hyper- and hypoautofluorescence). This is particularly the case when combining FAF modalities with different excitation wavelengths. In particular, the combination of short- with long-wavelength excitation FAF might be promising as it combines different penetration depths and excitation of different fluorophores and, hence, allows for detection and monitoring of (chorio)retinal lesions in different depths and of different biomolecular structures. Spectrally resolved FAF imaging might also represent a helpful addition as it allows for quantitative comparison of green and red emission fluorescent components [44]. However, it is important to note that FAF imaging alone is not sensitive enough to distinguish between different subtypes of posterior/panuveitis. Therefore, combination with other imaging modalities and laboratory testing remains the gold standard for diagnosis and exclusion of infectious etiologies, when suspected. Further, FAF has several limitations, including poor visualization in case of media opacity (cataract, vitreous opacities), an often-uncomfortable image acquisition for the patient (except for IRAF), and very limited options for quantification of the image signal. Moreover, clinical availability of FAF, especially IRAF, is limited.

FAF is a reliable method for detection of RPE atrophy, which can be more difficult to delineate with other imaging modalities [39]. FAF is not only useful for identification of damaged RPE, but also for monitoring of retinal lesions and scars [31]. It may be used as a marker of disease activity, as a monitor of the course of lesions and might also aid prediction of prognosis. Therefore, in selected uveitis entities, FAF may help to determine the adequate therapeutic approach, as it can aid coming to the correct diagnosis and differentiation of active from inactive disease [18,22,35,67,68,69,70,72]. Additionally, the use of ultra-wide-field FAF imaging can expand the detection of retinal and chorioretinal involvement within the periphery, which is otherwise difficult to monitor [72]. In addition to the literature on the application of FAF on distinctive uveitis entities, there is also evidence that FAF may aid evaluation of uveitis macular edema in general as increased central FAF is associated with poor vision [73].

Furthermore, combination of different non-invasive imaging methods (FAF modalities with different excitation wavelengths, OCT, CFP, infrared reflectance) for the monitoring of certain uveitis entities instead of recurrent invasive methods such as FA and ICG-A may be a useful alternative for routine clinical practice [66,74]. For example, in a case of chronic VKH syndrome, Vasconcelos-Santos et al. suggested the combination of FAF and OCT as a non-invasive alternative to FA and ICG-A to delineate RPE/PR changes and follow the progression of the disease [45]. The combination of FAF and OCT was also suggested by Yeh et al. to characterize and monitor damage of the RPE and retinal anatomy in PORN [54]. Whether FAF and other non-invasive methods such as OCT can replace gold-standard invasive methods such as FA and ICG-A needs further investigation, particularly in suspected macular neovascularization. In these cases, OCT angiography might represent another useful imaging addition [28]. Thus far, FA remains the gold standard for specific signs of active inflammation such as disk leak, retinal vascular leakage, or macular edema.

The strengths of this narrative review are that it covers a wide variety of different posterior and panuveitis entities, and that it followed a clear methodological approach which is explained in detail. Limitations include that it did not follow a full systematic approach and there were a relatively small number of subjects in some of the included articles; however, this reflects the very low prevalence of some posterior and panuveitis entities, emphasizing the need to generate further evidence for these rare diseases. This concerns, in particular, the rare conditions within the group of posterior uveitis, such as unilateral acute idiopathic maculopathy (UAIM) or acute retinal pigment epitheliitis (ARPE), for which we found no literature at all. Lastly, the current literature is sparse on findings using modalities with further excitations wavelengths apart from BAF (such as GAF or IRAF) and, hence, this overview lays its main focus on BAF.

In conclusion, FAF can aid diagnosing, distinction, and monitoring of different posterior and panuveitis entities, especially as part of a multimodal imaging approach. Further, in combination with such other non-invasive imaging techniques like OCT, CFP, infrared reflectance, and OCT angiography, it may also represent a non-invasive alternative to FA and ICG-A in some cases. Applicability of FAF in uveitis is even greater, when using a combination of short- and long-wavelength FAF; however, more research on the comparison of FAF with different excitation wavelengths and their diagnostic and monitoring value in posterior and panuveitis is warranted.

## Figures and Tables

**Table 1:** Overview of the different uveitis entities included in this review.

Non-Infectious	Infectious	Masquerade Syndromes
Acute posterior multifocal placoid pigment epitheliopathy (APMPPE)	Cytomegalovirus retinitis (CMV)	Intraocular lymphoma
Mulitple evanescent white dot syndrome (MEWDS)	Syphilis	Choroidal melanoma
Multifocal choroiditis and panuveitis (MCP)	Tuberculosis	
Punctate inner choroidopathy (PIC)	Acute retinal necrosis (ARN)	
Serpiginous choroiditis	Progressive outer retinal necrosis (PORN)	
Acute zonal occult outer retinopathy (AZOOR)		
Birdshot chorioretinopathy		
Vogt–Koyanagi–Harada (VKH) disease		
Behçet Uveitis		
Ocular Sarcoidosis		
